# Characteristics of* Escherichia coli* Isolated from Bovine Mastitis Exposed to Subminimum Inhibitory Concentrations of Cefalotin or Ceftazidime

**DOI:** 10.1155/2018/4301628

**Published:** 2018-11-01

**Authors:** Gang Liu, Laidi Ding, Bo Han, Sofie Piepers, S. Ali Naqvi, Herman W. Barkema, Tariq Ali, Sarne De Vliegher, Siyu Xu, Jian Gao

**Affiliations:** ^1^Department of Clinical Veterinary Medicine, College of Veterinary Medicine, China Agricultural University, Beijing, China; ^2^M-team and Mastitis and Milk Quality Research Unit, Department of Reproduction, Obstetrics, and Herd Health, Faculty of Veterinary Medicine, Ghent University, Ghent, Belgium; ^3^Department of Production Animal Health, Faculty of Veterinary Medicine, University of Calgary, Calgary, Canada

## Abstract

*Escherichia coli* is a major udder pathogen causing clinical mastitis in dairy cattle and its heat stable endotoxin in powdered infant formula milk is a potential risk factor in neonatal infections. Cephalosporins are frequently used for treatment of mastitis caused by mastitis; however, use of these antimicrobials may induce antimicrobial resistance in* E. coli*. The objective of this study was to explore the* in vitro* effect of subminimum inhibitory concentrations (sub-MIC) of cefalotin (CF) and ceftazidime (CAZ) on the morphology, antimicrobial resistance, and endotoxin releasing characteristics of 3* E. coli* isolates recovered from bovine clinical mastitis. The parent* E. coli* isolates, which were susceptible to CF and CAZ, were exposed to CF or CAZ separately at sub-MIC levels to produce 9 generations of induced isolates. Colonies of the CAZ-induced isolates from all 3 parent* E. coli *were smaller on blood agar and the bacteria became filamentous, whereas the CF-induced isolates did not demonstrate prominent morphological changes. After induction by CF or CAZ, many induced isolates showed resistance to cefoxitin, CAZ, CF, kanamycin, ampicillin, and amoxicillin/clavulanic acid while their parent isolates were susceptible to these antimicrobials. Notably, 5 CAZ-induced isolates from the same parent isolate were found to produce extended-spectrum beta-lactamase (ESBL) though none of the tested ESBL related genes could be detected. All CAZ-induced isolates released more endotoxin with a higher release rate, whereas endotoxin release of CF-induced* E. coli* isolates was not different from parent isolates. The exposure of cephalosporins at sub-MIC levels induced resistant* Escherichia coli. *We inferred that cephalosporins, especially CAZ, should be used prudently for treatment of clinical* E. coli* mastitis.

## 1. Introduction

Mastitis is one of the most common and costly diseases for the dairy industry worldwide. Mastitis also affects animal welfare and is a frequent reason that cows are culled [1]. Treatment of mastitis accounts for the majority of antimicrobials administered to dairy cows [2, 3]. Gram-negative bacteria, mostly coliforms, including* Escherichia coli*,* Klebsiella* spp., and* Enterobacter* spp., cause a high proportion of all clinical mastitis (CM) cases [4–6].* Escherichia coli* is the most common Gram-negative species causing CM in dairy cattle [4, 6, 7]. The endotoxin of* E. coli*, lipopolysaccharide (LPS), can remain biologically active in powdered infant formula milk because it is heat stable at 100°C and, therefore, may pose a potential risk to formula fed neonates [8].

The efficacy and necessity of antimicrobial therapy for treatment of* E. coli* mastitis are still a topic of debate [9]. However, fluoroquinolones and cephalosporins, particularly 3^rd^ and 4^th^ generation products, are the only antimicrobials for which there is supporting evidence of beneficial effects in treatment of* E. coli* mastitis [10, 11]. It is noteworthy that these 2 classes of antimicrobial agents are also important drugs for human health. The prevalence of resistance against these important antibiotics, particularly in* E. coli*, is increasing worldwide in veterinary and human medicine [12–14]. The 2 main factors involved in development of antimicrobial resistance in bacteria are the presence of resistance genes and selective pressure of antimicrobial agents, especially if suboptimal doses are administered [15]. Currently, very limited studies have been conducted to elucidate the influence of cephalosporin dosage on induction of antimicrobial resistance in* E. coli* isolated from CM. First-generation cephalosporins are less critical for human health, although they primarily have a Gram-positive spectrum, with limited activity against Gram-negative bacteria [16, 17].

Induction of antimicrobial resistance in* E. coli* has been characterized by changes in morphology, including filamentation, which is likely to protect the bacteria from deleterious effects of antimicrobials [18, 19]. There is also strong evidence that antimicrobials may enhance endotoxin release from* E. coli*, which could exacerbate symptoms of CM [18].

The main objective of this study was, therefore, to determine* in vitro* effects of sub-MIC exposure of cefalotin (CF) or ceftazidime (CAZ) on 3* E. coli *isolates recovered from bovine CM cases. Characteristics such as colony morphology, antimicrobial susceptibility profile, and release of endotoxin from* E. coli* isolates recovered after induction were investigated.

## 2. Materials and Methods

### 2.1. Escherichia coli Isolates

Quarter milk samples (n=1252) were collected from July 2015 to May 2016 from dairy cows with CM from various dairy herds located in 16 Chinese provinces [7]. In total, 153* E. coli* isolates were recovered, of which 36 produced extended-spectrum beta-lactamase (ESBL). More details on the origin and characteristics of the* E. coli* isolates are described [12]. All isolates were tested for resistance to CF (30 *μ*g) and CAZ (30 *μ*g) using the Kirby-Bauer disk diffusion method, with clinical breakpoints following recommendations of the Clinical and Laboratory Standards Institute [20]. Three* E. coli* isolates, subsequently referred to as E4, E11, and E21, were selected from 3 provinces (Beijing, Shanghai, and Gansu) and used as parent* E. coli* isolates in this study. The 3 isolates were susceptible to CF and CAZ, did not produce ESBL, and did not carry any of the tested ESBL encoding genes (data not shown).

### 2.2. Experimental Design

All steps of the experiment were carried out in triplicate. A single colony was inoculated into 20 mL trypticase soy broth (TSB; BD, Franklin Lake, NJ) and incubated for 12 h at 37°C. Then, 200 *μ*L of the bacterial suspension (0.5 McFarland) was inoculated into 20 mL Mueller-Hinton broth (Luqiao, Beijing, China) to which 1/2 of the MIC was added for CF and CAZ separately and incubated for 24 h at 37°C. Subsequently, the bacterial suspension was centrifuged (6000 ×* g*, 25°C, 5 min) (Anting, Shanghai, China) and the pellet resuspended in a solution containing 1.4 mL TSB and 0.6 mL glycerol (Luqiao, Beijing, China) and stored pending further processing. These induced isolates (i.e., first generation of growth) are further referred to as CF-1 groups (E4-CF-1, E11-CF-1, and E21-CF-1). The MIC values for CF versus CF-1 groups were determined, and 1/2 of the MIC of CF was again dissolved in Mueller-Hinton broth (Luqiao, Beijing, China) containing 1.5 x 10^6^ CFU/mL of CF-1 groups. After 24 h of incubation at 37°C, the 2^nd^ generation induced mutant was obtained and identified as CF-2 group. The same steps were repeated another 7 times. Induced* E. coli* isolates recovered after subsequent induction were designated CF-3, CF-4, CF-5, CF-6, CF-7, CF-8, and CF-9.* Escherichia coli* isolates were recovered after induction with CAZ, using procedures identical to those mentioned above for CF. Isolates recovered after induction were identified as CAZ-1, CAZ-2, CAZ-3, CAZ-4, CAZ-5, CAZ-6, CAZ-7, CAZ-8, and CAZ-9 (E4-CAZ, E11-CAZ, and E21-CAZ). Parent isolates and all induced isolates recovered after induction were stored in 30% glycerol at -80°C pending further processing. When MICs of the parent* E. coli* and induced isolates were determined,* E. coli* ATCC 25922 was used as a control strain. The CLSI breakpoints for CF were ≤ 8 *μ*g/mL (susceptible), 16 *μ*g/mL (intermediate), and ≥ 32 *μ*g/mL (resistant). In case of CAZ, breakpoints for ceftiofur (≤ 2 *μ*g/mL = susceptible, 4 *μ*g/mL = intermediate, and ≥ 8 *μ*g/mL = resistant) were used [20].

### 2.3. Morphological Observations

Stored isolates were recovered by streaking 100 *μ*L of suspension on tryptone soya agar (TSA; Luqiao, Beijing, China) supplemented with 5% sheep blood and incubated for 24 h at 37°C. A pure growth was picked and streaked on a new TSA plate. Shape, size, and color of colonies were recorded. A single colony from agar was stained using the Gram-staining method and morphology examined with an optical microscope (Olympus, Tokyo, Japan).

### 2.4. Antimicrobial Susceptibility Testing

Antimicrobial susceptibility of the parent* E. coli* and isolates recovered after induction was determined using Mueller-Hinton agar (BD, Franklin Lakes, NJ) against 9 antimicrobial agents using the standard Kirby-Bauer disk diffusion method according to CLSI recommendations [20]. Inhibition zone diameter (mm) was measured using a ruler. The panel of antimicrobial agents consisted of ampicillin (10 *μ*g), amoxicillin/clavulanic acid (20/10 *μ*g), CAZ (30 *μ*g), CF (30 *μ*g), cefepime (30 *μ*g), cefoxitin (30 *μ*g), gentamicin (10 *μ*g), kanamycin (30 *μ*g), and amikacin (30 *μ*g). For these antimicrobial agents, breakpoints for cefepime and cefoxitin referred to* E. coli* isolates from humans.* Escherichia coli* ATCC 25922 and* Enterobacter cloacae* CMCC45301 were used as quality control strains.

### 2.5. Detection of ESBL Production

Parent isolates and all isolates recovered after induction were screened on MacConkey agar containing cefotaxime (1 mg/L) for ESBL-production. Presumptive ESBL-producing isolates, if any, were further confirmed by the double-disc synergy test, following recommendations of the CLSI [20], using antimicrobial discs of cefotaxime (30 *μ*g), cefotaxime plus clavulanic acid (30/10 *μ*g), CAZ (30 *μ*g), and CAZ plus clavulanic acid (30/10 *μ*g). Production of ESBL was considered positive if the inhibition zone of cefotaxime plus clavulanic acid or CAZ plus clavulanic acid was ≥ 5 mm larger than their respective single discs [20].* Escherichia coli* ATCC 25922 (ESBL-negative strain) and* Klebsiella pneumoniae* ATCC 700603 (ESBL-positive strain) were used as reference strains.

### 2.6. Detection of ESBL-Related Genes

Bacterial DNA from* E. coli* was isolated using the Bacterial DNA Extraction Kit (Transgen, Beijing, China) according to the manufacturer's instructions. A PCR assay was conducted to detect the presence of *bla*_CTX-M_, *bla*_CTX-M-1_, *bla*_CTX-M-2_, *bla*_CTX-M-9_, *bla*_CTX-M-15_, *bla*_SHV_, *bla*_TEM_, and *bla*_OXA_ genes, as described [21, 22] with minor modifications. The reaction mixture (20 *μ*L) consisted of 10 *μ*L of TaqMix (Transgen, Beijing, China), 1 *μ*L of template DNA, 0.5 *μ*L of each primer (10 *μ*M; Sunbiotech, Beijing, China), and 8 *μ*L of ultra-pure distilled water. Initial denaturation at 94°C for 5 min was followed by 35 cycles of amplification at 94°C for 45 s, annealing at 55°C for 30 s, and extension at 72°C for 60 s, and a final step with extension at 72°C for 10 min. The PCR products were separated on a 2% agarose gel. Subsequently, PCR products were purified by TIANquick Midi Purification Kit (TIANGEN, Beijing, China) and then bidirectionally sequenced using the same primers by ABI 3730 sequencer (Applied Biosystems, Foster City, CA). Gene sequences were aligned with BLASTN software (http://www.ncbi.nlm.nih.gov/BLAST/) and compared to sequences available in GenBank.* Klebsiella pneumoniae* ATCC 700603 (ESBL-positive strain) and ddH_2_O, without template DNA, were used as positive and negative controls, respectively, in all PCR assays. Primers used in this study are presented in Table 1.

### 2.7. Detection of Endotoxin Release

Isolates recovered after induction with CF or CAZ, as well as parent isolates, were used for this part of the study. Frozen bacteria were thawed at 37°C for 60 min. Ten *μ*L of suspension was streaked onto TSA supplemented with 5% sheep blood and incubated for 24 h at 37°C. A single colony was picked from the agar, inoculated into 10 mL of TSB, and incubated for 18 h at 37°C. Bacterial counts were measured using the 10-fold dilution method. Subsequently, bacterial solutions were diluted to 10^6^ CFU/mL using TSB and bacteria were cultured aerobically at 37°C. At various time points (0, 2, 4, 6, 8, and 10 h), aliquots were collected and endotoxin release capability quantified. Briefly, aliquots were collected and centrifuged at 6,000 ×* g *for 15 min. The supernatant of each aliquot was collected and stored at -20°C until further processing. Endotoxin concentration was measured using an enzyme-linked immunosorbent assay (ELISA) commercial kit (Sigma, St. Louis, MO). Each step was performed following the manufacturer's instructions. All experiments were performed in triplicate.

### 2.8. Statistical Analyses

Associations between endotoxin release and exposure to sub-MICs of CF and CAZ were determined by fitting a mixed effects linear regression model using replicate (n = 3) as the random effect with the lme4 package (Bates et al., 2017) in R version 3.3.0 (R Core Team). A* P-*value < 0.05 was considered statistically significant. Data were first visually examined to understand associations between time and endotoxin release. Due to the sigmoidal nature of the association, data were normalized by taking the natural logarithm of endotoxin release, which resulted in a more clearly parabolic association between incubation time and endotoxin release. Higher degree terms for time were included to more accurately adjust for this parabolic relationship and isolate effects of treatment and generation of isolate on endotoxin release. The final model included treatment (CF, CAZ, or control) and an interaction with generation (ranging from 1 to 9) as independent variables, with an adjustment for incubation time modelled with cubic and quadratic terms, as higher degree polynomial terms did not increase model fit as determined by* P*-value and change in restricted maximum likelihood criterion.

## 3. Results

### 3.1. MICs of Cefalotin and Ceftazidime

The MICs of CF and CAZ for the parent* E. coli* isolates were 0.5-4 *μ*g/mL and 0.125-1 *μ*g/mL, respectively; therefore, parent isolates were susceptible to these 2 antimicrobial agents. The MIC values of CF and CAZ against induced isolates are shown (Table 2). After exposure to sub-MIC of CF, 8, 7, and 6 isolates that were derived from E4, E11, and E21, respectively, were resistant to CF. After exposure to sub-MIC of CAZ, 6, 8, and 6 isolates derived from E4, E11, and E21, respectively, were resistant to CAZ.

### 3.2. Morphological Characteristics

**Figure 1:**
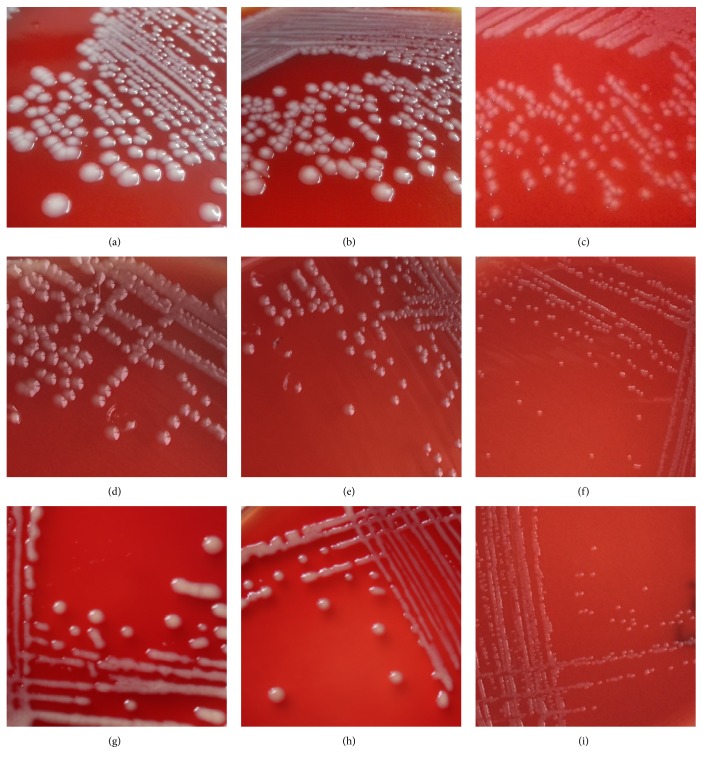
Colony morphology of parent* Escherichia coli* and antibiotic-induced isolates recovered after induction (24 h). (a), (d), and (g) were parent strains; (b), (e), and (h) were cefalotin-induced isolates; and (c), (f), and (i) were ceftazidime-induced isolates.

**Figure 2:**
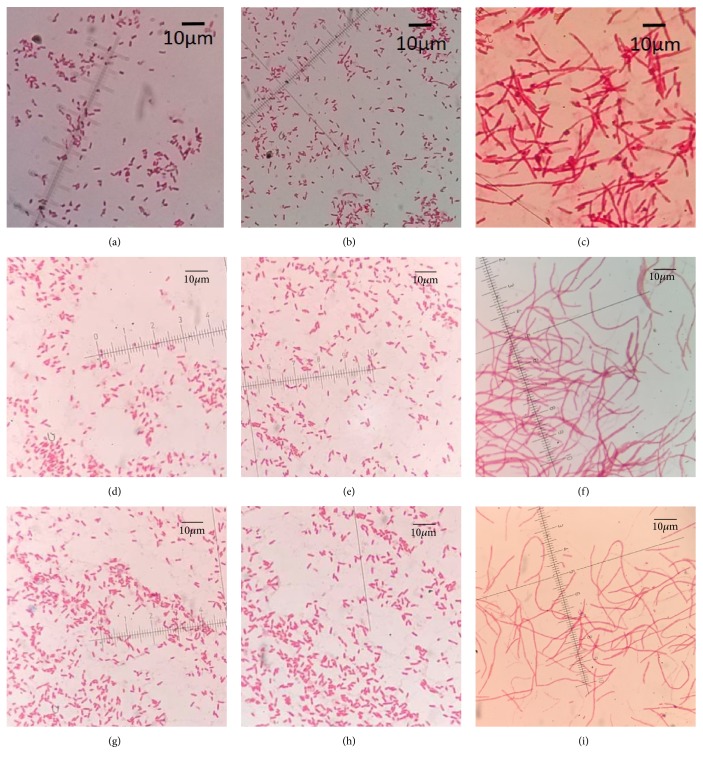
Cell morphology of parent* Escherichia coli* and isolates recovered after induction with antibiotics. Cells were subjected to Gram-staining and examined with an optical microscope (1000×). (a), (d), and (g) were parent strains; (b), (e), and (h) were cefalotin-induced isolates; and (c), (f), and (i) were ceftazidime-induced isolates.

Morphological characteristics of* E. coli* isolates recovered after induction with CF were similar to those of their parent isolates (Figures 1 and 2). However, morphological changes on blood agar were observed in 17 isolates recovered after induction with CAZ compared to their parent isolates. Of those isolates, 6, 5, and 6 were derived from E4, E11, and E 21, respectively. Colonies of those isolates were smaller after 24 h incubation at 37°C and became sticky and were therefore difficult to retrieve (Figure 1). Also, cells of all isolates recovered after induction with CAZ were elongated, with a filamentous shape (Figure 2).

### 3.3. Antimicrobial Susceptibility Profiles

Among CF-induced isolates, 17, 13, and 13 developed intermediate resistance to cefoxitin, ceftazidime, and kanamycin, respectively, whereas no isolate was resistant to these 3 antimicrobial agents (Table 3). Of isolates with intermediate resistance to cefoxitin, 7, 5, and 5 were derived from E4, E11, and E21, respectively. Of isolates with intermediate resistance to kanamycin, 6, 3, and 4 were derived from E4, E11, and E21, respectively. In addition, 6 and 4 isolates derived from E4 and E21 were resistant to ampicillin and amoxicillin/clavulanic acid, respectively, whereas 7 and 6 isolates derived from E11 became resistant to ampicillin and amoxicillin/clavulanic acid. All CF-induced isolates were susceptible to cefepime, gentamicin, and amikacin. Parent isolates remained susceptible to all tested antimicrobial agents.

Among CAZ-induced isolates, all were resistant to CF. Nineteen isolates became resistant to cefoxitin, of which 6, 7, and 6 were derived from E4, E11, and E21, respectively. Only 1 CAZ-induced isolate (CAZ-1 derived from E4) was susceptible to ampicillin and amoxicillin/clavulanic acid. All isolates derived from E4 and E21 became intermediate to kanamycin, whereas CAZ-1 from E11 remained susceptible (Table 4). All CAZ-induced isolates were susceptible to cefepime, gentamicin, and amikacin. Parent isolates were susceptible to all tested antimicrobial agents.

### 3.4. ESBL Production and ESBL-Related Genes

**Figure 3:**
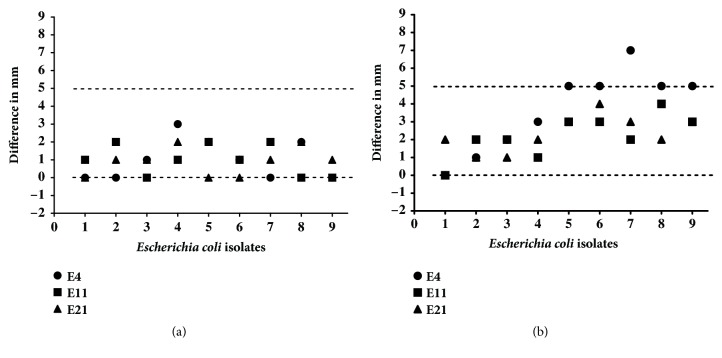
Extended spectrum beta-lactamase (ESBL) production of isolates recovered after induction by cefalotin (a) or ceftazidime (b). Isolates at various generations are listed on the X-axis. Differences were calculated as diameter of the inhibition zone of ceftazidime plus clavulanic acid subtracted from their respective single discs. An isolate was designated an ESBL producer if the difference was > 5 mm.

The difference in diameters between the inhibition zone of cefotaxime and cefotaxime plus clavulanic acid was < 5 mm for all induced isolates, except 5 derived from E4 (CAZ-5 to CAZ-9). The difference of the zone diameters for those 5 CAZ-induced isolates was ≥ 5 mm (Figure 3), which was an indication for ESBL production by those isolates. However, none of the tested ESBL-encoding genes were detected in ESBL-producing isolates.

### 3.5. Endotoxin Release

**Figure 4:**
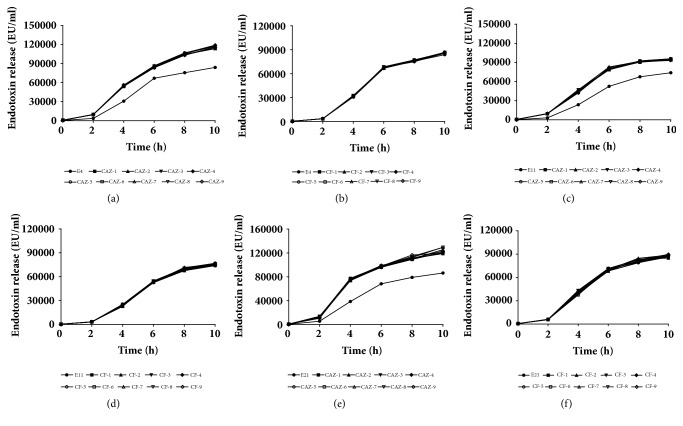
Endotoxin release of* Escherichia coli* isolates included in the study at various time points (0, 2, 4, 6, 8, and 10 h).

Endotoxin release increased with time (2, 4, 6, 8, and 10 h) for 2 treatment types and control (*P *< 0.0001). The amount of endotoxin released by CAZ-induced mutants differed from the parent* E. coli* isolates (*P *< 0.001). Therefore, CAZ at sub-MIC increased release of endotoxin from induced* E. coli* mutants when compared to parent strains (Figure 4). However, the amount of endotoxin released by CAZ mutants was not associated with generation of the isolate (*P = *0.94). There was no difference in amount of endotoxin released at the same time point for CF-induced mutants compared to parent* E. coli* isolates (*P *= 0.70), nor did endotoxin release by CF-mutants vary by generation (*P = *0.79). Therefore, CF at sub-MIC did not increase endotoxin release, compared to parent isolates, at any time point.

Both antibiotic type and incubation time were associated with endotoxin release (Table 5; Figure 4). All treatments followed similar release patterns over time, starting with a high rate of endotoxin release that plateaued after 6 h.

## 4. Discussion

This was apparently the first study to characterize changes in morphology, antimicrobial resistance, and endotoxin releasing of* E. coli* isolated from bovine CM under persistent exposure to CF and CAZ at sub-MIC. In this study, sub-MIC* in vitro* exposure of CF and CAZ resulted not only in failure of killing* E. coli*, but also in increased antimicrobial resistance. In addition, exposure to CAZ led to generation of more virulent strains of* E. coli*. Choice of antibiotic, administration route, and treatment duration are determined by many factors [23], which makes the choice of treatment strategy relatively subjective or arbitrary. Based on our findings, we inferred that prudent usage of antibiotics should be proposed when treating CM, particularly if caused by* E. coli*.

After continuous sub-MIC exposure to CF or CAZ, isolates became increasingly resistant to CF or CAZ, respectively. Bacteria can quickly develop resistant phenotypes under persistent cephalosporin stress [24]. In addition, several isolates became resistant to *β*-lactams (CF, cefoxitin, CAZ, ampicillin, and amoxicillin/clavulanic acid) after exposure to either CF or CAZ. Similarly, bovine* E. coli* strains isolated from cows treated with ceftiofur became resistant to cefazoline, whereas strains not previously exposed to ceftiofur remained susceptible [25]. Resistance of* E. coli* strains to multiple antimicrobials could result in failure of mastitis therapy and is a serious threat to human and animal health. Mutants of* Pseudomonas aeruginosa* isolated from humans became resistant to various antimicrobials after induction with ceftazidime [26]. Resistance to vancomycin increased in* Staphylococcus aureus* isolates, accompanied with morphologic alteration, after continuous exposure to ceftazidime [27]. Results of these 2 studies were in accordance with our findings.

Colonies of* E. coli* isolates recovered after induction with CAZ were smaller and stickier than the parent isolate. This was in accordance with a previous study that growth of* Bacteroides thetaiotaomicron* isolates was slower after exposure to cefoxitin [28]. In our study, bacterial cells became extensively elongated (filamentation), consistent with several* E. coli* strains that become filamentous after exposure to various antimicrobial agents [18, 19, 29].* Escherichia coli* readily became filamentous in shape when exposed to cephalosporins [29]. Effects of antibiotics on bacterial morphology or survival depend on both type and dosage [19, 30]. Antibiotics can kill bacteria by acting on penicillin-binding proteins (PBPs) [31]. There are 3 kinds of PBPs: PBP1, PBP2, and PBP3. Various types and dosages of antibiotics could bind to different PBPs, which may result in a morphology change of bacteria [30]. A high concentration of CAZ can destroy bacteria quickly by inhibiting PBP1, whereas the same antibiotic administered at lower concentrations can lead to filamentation of bacteria [32]. In the present study, filamentation of isolates at 6^th^ and later generations of growth was weaker than during earlier generations; perhaps induced isolates were already adapted to antimicrobial exposure. It has been reported that the filamentous bacteria return to a typical bacillus shape after antibiotic removal, so filamentation was temporary [33]. Interestingly, isolates recovered after induction with CF did not undergo obvious changes in colonial and bacterial morphology. Presumably, CF bound to different PBPs, which did not make the cell filamentous.

In this study, 5 isolates recovered after induction with CAZ produced ESBL phenotype, while no ESBL-associated genes were detected in these isolates. One potential reason for this discrepancy might be that we were unable to detect the genes that were responsible for ESBL production. In the present study, we only tried to detect common ESBL-encoding genes, as described [21, 22]. Nevertheless, other ESBL-encoding genes not targetted in this study may have been responsible for production of ESBL [34]. Moreover, there might be a misinterpretation of data, since the difference of 4 of 5 isolates was just at the threshold (5 mm) to detect ESBL phenotypes.

Release of endotoxins from the disrupted* E. coli* cell walls could cause a serious inflammatory reaction in an infected udder [35]. Besides, high endotoxin in ingested infant formula milk might cause neonatal bacteraemia and endotoxemia, especially in neonates with immature innate immune systems [8]. In our study, all parent and induced isolates released more endotoxin when incubation was prolonged, which was in accordance with reports that bacteria released more endotoxin in induction period by antibiotics due to overexpression or shedding at higher speed from cell wall [36, 37]. Increased release of endotoxin from isolates recovered after induction with CAZ may have been associated with filamentation of* E. coli* after exposure to CAZ [19, 30]. Filamentation of cells could increase the surface of the cell wall with a higher endotoxin release from cell wall and, therefore, a higher releasing speed. In contrast, isolates recovered after induction with CF did not produce more endotoxin than parent isolates, which may be associated with less morphological changes in these isolates.

Although a series of phenotypic changes (morphology, antimicrobial resistance, and endotoxin releasing capability) were observed in the current study, mechanism of bacteria's acquired resistance after exposure to the antibiotics should be further clarified. Therefore, a study including whole-genome sequencing and analysis of acquired mutations should be conducted, to provide insights regarding the origin of observed resistance.

## 5. Conclusions

After exposure to subminimum concentrations of CF or CAZ, parent* E. coli* CM isolates underwent a series of changes related to morphology, antimicrobial resistance pattern, and endotoxin release. Several isolates recovered after induction had higher resistance to CF, CAZ, and some other commonly used antimicrobials. Moreover, several isolates recovered after induction with CAZ had slower growth on blood agar, became filamentous, and released more endotoxin. Therefore, we inferred that there shoud be prudent use of cephalosporins, especially those that belong to the 3^rd^ generation, for treatment of bovine mastitis caused by* E. coli*. Such treatment would increase potential risk to formula fed neonates if the milk with endotoxin was processed into powdered infant formula milk.

## Figures and Tables

**Table 1:** Primers used to detect extended spectrum beta-lactamase encoding genes.

Genes	Primer sequence (5′ to 3′)	Amplicon size (bp)
*bla*_CTX-M_	CGC TTT GCG ATG TGC AG	550
	ACC GCG ATA TCG TTG GT	
*bla*_CTX-M-1_	GTT ACA ATG TGT GAG AAG CAG	1041
	CCG TTT CCG CTA TTA CAA AC	
*bla*_CTX-M-2_	ATG ATG ACT CAG AGC ATT CGC CGC	876
	TCA GAA ACC GTG GGT TAC GAT TTT	
*bla*_CTX-M-9_	GTG ACA AAG AGA GTG CAA CGG	857
	ATG ATT CTC GCC GCT GAA GCC	
*bla*_CTX-M-15_	CAC ACG TGG AAT TTA GGG ACT	995
	GCC GTC TAA GGC GAT AAA CA	
*bla*_TEM_	TTC TTG AAG ACG AAA GGG C	1150
	ACG CTC AGT GGA ACG AAA AC	
*bla*_SHV_	CAC TCA AGG ATG TAT TGT G	885
	TTA GCG TTG CCA GTG CTC G	
*bla*_OXA_	ACA CAA TAC ATA TCA ACT TCG C	813
	AGT GTG TTT AGA ATG GTG ATC	

**Table 2:** Minimal inhibitory concentrations of cefalotin and ceftazidime against *Escherichia coli* isolates.

		MIC of *E. coli* isolates (*μ*g/mL)									
Isolate	Antimicrobial	Parent type	-1_ _^*∗*^	-2	-3	-4	-5	-6	-7	-8	-9
E4	Cefalotin†	4	8	32_ _^#^	80_ _^#^	240_ _^#^	240_ _^#^	320_ _^#^	320_ _^#^	320_ _^#^	320_ _^#^
	Ceftazidime‡	0.125	0.25	2	4	16_ _^#^	16_ _^#^	32_ _^#^	32_ _^#^	64_ _^#^	128_ _^#^
E11	Cefalotin	0.5	2	16	32_ _^#^	#	#	#	#	#	#
	Ceftazidime	1	4	8_ _^#^	8_ _^#^	16_ _^#^	32_ _^#^	32_ _^#^	64	128_ _^#^	128_ _^#^
E21	Cefalotin	2	8	16	16	#	#	#	#	#	#
	Ceftazidime	0.5	1	4	4	8_ _^#^	16_ _^#^	16_ _^#^	32_ _^#^	32_ _^#^	64_ _^#^

No mark: susceptible or intermediate to cefalotin or ceftazidime; #: resistant to cefalotin or ceftazidime. Breakpoints for cefalotin were as follows: susceptible ⩽ 8 *μ*g/mL, intermediate 16 *μ*g/mL, and resistant *⩾* 32 *μ*g/mL. Breakpoints for ceftazidime were as follows: susceptible ⩽ 2 *μ*g/mL, intermediate 4 *μ*g/mL, and resistant *⩾* 8 *μ*g/mL.

^*∗*^*Escherichia coli* isolates at various generations that were induced by cefalotin or ceftazidime.

†MIC values of cefalotin were determined for *E. coli* isolates induced by cefalotin.

‡MIC values of ceftazidime were determined for *E. coli* isolates induced by ceftazidime.

**Table 3:** Antimicrobial susceptibility profiles of the* Escherichia coli* isolates recovered after induction with cefalotin.

	Resistant *E. coli* isolates		
Antimicrobial	Derived from E4	Derived from E11	Derived from E21
Cefoxitin	CF-3/-4/-5/-6/-7/-8/-9	CF-5/-6/-7/-8/-9	CF-5/-6/-7/-8/-9
Ceftazidime	CF-5/-6/-7/-8/-9	CF-5/-6/-7/-8/-9	CF-7/-8/-9
Ampicillin	CF-4/-5/-6/-7/-8/-9	CF-3/-4/-5/-6/-7/-8/-9	CF-6/-7/-8/-9
Amoxicillin/clavulanic acid	CF-4/-5/-6/-7/-8/-9	CF-4/-5/-6/-7/-8/-9	CF-6/-7/-8/-9
Kanamycin_ _^*∗*^	CF-4/-5/-6/-7/-8/-9	CF-7/-8/-9	CF-6/-7/-8/-9

^*∗*^All *E. coli* isolates had intermediate resistance to cefoxitin, ceftazidime, and kanamycin.

**Table 4:** Antimicrobial susceptibility profiles of the *Escherichia coli* isolates recovered after induction with ceftazidime.

	Resistant *E. coli* isolates		
Antimicrobial	Derived from E4	Derived from E11	Derived from E21
Cefalotin	CAZ-1/-2/-3/-4/-5/-6/-7/-8/-9	CAZ-1/-2/-3/-4/-5/-6/-7/-8/-9	CAZ-1/-2/-3/-4/-5/-6/-7/-8/-9
Cefoxitin	CAZ-4/-5/-6/-7/-8/-9	CAZ-3/-4/-5/-6/-7/-8/-9	CAZ-4/-5/-6/-7/-8/-9
Ampicillin	CAZ-2/-3/-4/-5/-6/-7/-8/-9	CAZ-1/-2/-3/-4/-5/-6/-7/-8/-9	CAZ-1/-2/-3/-4/-5/-6/-7/-8/-9
Amoxicillin/ clavulanic acid	CAZ-2/-3/-4/-5/-6/-7/-8/-9	CAZ-1/-2/-3/-4/-5/-6/-7/-8/-9	CAZ-1/-2/-3/-4/-5/-6/-7/-8/-9
Kanamycin_ _^*∗*^	CAZ-1/-2/-3/-4/-5/-6/-7/-8/-9	CAZ-2/-3/-4/-5/-6/-7/-8/-9	CAZ-1/-2/-3/-4/-5/-6/-7/-8/-9

^*∗*^All *E. coli* isolates had intermediate resistance to kanamycin.

**Table 5:** Results of a linear mixed regression model for log endotoxin release (log of EU/mL) by *Escherichia coli* after exposure to cefalotin (CF) or ceftazidime (CAZ).

Parameter	Estimate	SEM	*P*-value
Parent isolates (Ref.)_ _^*∗*^	6.07	0.11	<0.001
CAZ	0.50	5.01 x 10^−3^	<0.001
CF	0.02	5.01 x 10^−3^	0.699
Interactions†			
CAZ x generation	3.78 x 10^−4^	5.23 x 10^−3^	0.942
CF x generation	1.39 x 10^−3^	5.23 x 10^−3^	0.791
Time (h)	1.60	0.02	<0.001
Time^2^ (h^2^)	-0.16	5.39 x 10^−3^	<0.001
Time^3^ (h^3^)	0.01	3.54 x 10^−4^	<0.001

Between-replicate variance	0.03	0.16	

Endotoxin release was measured at various time points (2, 4, 6, 8, and 10 h) and resistance was induced over varying numbers of generations.

^*∗*^Baseline to which other samples were compared was a parent isolate (generation 0)

†Interactions were not created with the parent isolate as there was only 1 generation

## Data Availability

The data used to support the findings of this study are available from the corresponding author upon request.
